# Supplementary material for: Cost-effectiveness of percutaneous patent foramen ovale closure versus medical therapy for cryptogenic stroke prevention: A Chinese healthcare perspective
Source: PLoS One. 2026 Mar 17;21(3):e0345015. doi: 10.1371/journal.pone.0345015 (PMC12994829; doi:10.1371/journal.pone.0345015)
Supplement: S1 Checklist — Consolidated Health Economic Evaluation Reporting Standards (CHEERS) 2022 checklist for reporting health economic evaluations. (DOCX) [file pone.0345015.s001.docx]

# CHEERS 2022 Checklist

## Consolidated Health Economic Evaluation Reporting Standards (CHEERS) 2022

****Study Title:**** Cost-Effectiveness of Percutaneous Patent Foramen Ovale Closure versus Medical Therapy for Cryptogenic Stroke Prevention: A Chinese Healthcare Perspective

| **Section** | **Item No.** | **Item** | **Reported (Yes/No)** | **Page/Line No.** |
| --- | --- | --- | --- | --- |
| ****Title and Abstract**** |  |  |  |  |
| Title | 1 | Identify the study as an economic evaluation and specify the interventions being compared | Yes | Title Page, Line 2-4 |
| Abstract | 2 | Provide a structured summary including objectives, perspective, setting, methods, results, and conclusions | Yes | Lines 38-67 |
| ****Introduction**** |  |  |  |  |
| Background and objectives | 3 | Provide an explicit statement of the broader context for the study, present the study question and its relevance to health policy or practice decisions | Yes | Lines 68-101 |
| ****Methods**** |  |  |  |  |
| Health economic analysis plan | 4 | Indicate whether a health economic analysis plan was developed and where available | No | N/A |
| Study population | 5 | Describe characteristics of the study population (e.g., age range, demographics, socioeconomic, or clinical characteristics) | Yes | Lines 162-168 |
| Setting and location | 6 | Provide relevant contextual information that may influence findings (e.g., country, healthcare system characteristics) | Yes | Lines 102-110, 195-203 |
| Comparators | 7 | Describe the interventions or strategies being compared and why chosen | Yes | Lines 46-48, 98-101 |
| Perspective | 8 | State the perspective(s) adopted by the study and why chosen | Yes | Lines 48-49, 196-197 |
| Time horizon | 9 | State the time horizon for the study and why appropriate | Yes | Lines 149-161 |
| Discount rate | 10 | Report the discount rate(s) used for costs and outcomes and why chosen | Yes | Lines 160-161 |
| Selection of outcomes | 11 | Describe what outcomes were used as the measure(s) of benefit and why selected | Yes | Lines 51-53, 271-277 |
| Measurement of outcomes | 12 | Describe how outcomes used to capture benefit were measured | Yes | Lines 252-270 |
| Valuation of outcomes | 13 | Describe the population and methods used to measure and value outcomes | Yes | Lines 253-270 |
| Measurement and valuation of resources and costs | 14 | Describe how costs were valued | Yes | Lines 195-233 |
| Currency, price date, and conversion | 15 | Report the dates of the estimated resource quantities and unit costs, describe methods for adjusting estimated unit costs to the year of reported costs if necessary, describe methods for converting costs into a common currency base and the exchange rate | Yes | Lines 196-197 |
| Rationale and description of model | 16 | If modelling is used, describe the type of model used, why this type of model was chosen, and key structural assumptions | Yes | Lines 112-143 |
| Analytics and assumptions | 17 | Describe any methods for analysing or statistically transforming data, any extrapolation methods, and approaches for validating the model | Yes | Lines 144-145, 178-181, 309-314 |
| Characterizing heterogeneity | 18 | Describe any methods used for estimating how the results of the study vary for subgroups | Yes | Lines 404-414 |
| Characterizing distributional effects | 19 | Describe how distributional effects were considered | Partial | Lines 411-414 |
| Characterizing uncertainty | 20 | Describe methods to characterize uncertainty in the analysis | Yes | Lines 278-308 |
| Approach to engagement with patients and others affected by the study | 21 | Describe any approaches to engage patients or service recipients, the general public, communities, or stakeholders in the design of the study | No | N/A |
| ****Results**** |  |  |  |  |
| Study parameters | 22 | Report all analytic inputs (e.g., values, ranges, references) including uncertainty or distributional assumptions | Yes | Tables 1-2, Lines 169-270 |
| Summary of main results | 23 | Report mean values for the main categories of costs and outcomes of interest and summarize them in the most appropriate overall measure | Yes | Lines 316-343, Tables 3-4 |
| Effect of uncertainty | 24 | Describe how uncertainty about analytic judgments, inputs, or projections affect findings. Report the effect of choice of discount rate and time horizon, if applicable | Yes | Lines 348-403, Table 5 |
| Effect of engagement with patients and others affected by the study | 25 | Report any insights from patients and others affected by the study that were considered in the analysis | No | N/A |
| ****Discussion**** |  |  |  |  |
| Study findings, limitations, generalizability, and current knowledge | 26 | Report key findings, limitations, ethical or equity considerations not captured, and how these could affect patients, policy, or practice | Yes | Lines 438-519 |
| ****Other Relevant Information**** |  |  |  |  |
| Source of funding | 27 | Describe how the study was funded and any role of the funder in the identification, design, conduct, and reporting of the analysis | Partial | Line 21 (Funding acquisition: XY) |
| Conflicts of interest | 28 | Report authors' conflicts of interest according to journal or ICMJE requirements | Yes | Lines 33-34 |
