# Supplementary material for: Cost-effectiveness of percutaneous patent foramen ovale closure versus medical therapy for cryptogenic stroke prevention: A Chinese healthcare perspective
Source: PLoS One. 2026 Mar 17;21(3):e0345015. doi: 10.1371/journal.pone.0345015 (PMC12994829; doi:10.1371/journal.pone.0345015)
Supplement: Attachment — Submitted filename: Commnets.docx [file pone.0345015.s006.docx]

**Cost-Effectiveness of P 40 ercutaneous Patent Foramen Ovale Closure versus Medical Therapy for Cryptogenic Stroke Prevention: A Chinese Healthcare Perspective**

**Comments**

1. The abstract summarizes the key findings effectively; however, it would benefit from explicitly identifying the sources of clinical efficacy data, such as specific trials like RESPECT, to offer immediate context for the transition probabilities utilized in the model.
2. The introduction offers a comprehensive overview of cryptogenic stroke and the prevalence of PFO in China, underpinned by pertinent statistics. Nonetheless, it does not address potential confounders such as RoPE score or anatomical PFO characteristics, which may affect patient selection and generalizability.
3. The Markov model comprising four health states (stable, post-minor stroke, post-moderate stroke, death) effectively captures long-term outcomes. However, the model presumes that there are no transitions back to a stable state following recurrent strokes, potentially oversimplifying the dynamics of recovery in real-world contexts.
4. The selection of a 30-year horizon and 3-month cycles is appropriate; however, the half-cycle correction is referenced without elaboration on its application in the results. Validation against shorter horizons in the base case may enhance the robustness of claims.
5. Transition probabilities are mainly based on data from the RESPECT trial, which provides high-quality evidence. Relying predominantly on a single Western trial for Chinese patients raises concerns regarding external validity; integrating data from Chinese registries, such as the 2021 registry, could provide a more accurate representation of local recurrence rates.
6. Cost parameters are gathered from Chinese hospital surveys and publications, thereby improving context-specificity. The reliance on expert opinion for estimating psychological comorbidity costs, such as depression and anxiety at $125 quarterly, presents a significant concern due to the absence of empirical support. This factor notably impacts sensitivity results, warranting a more rigorous justification or testing of exclusion methods.
7. Utility values are derived from Chinese studies; however, the post-PFO closure utilities (0.84 initial, 0.88 long-term) presuppose enhancements attributed to decreased anxiety, lacking direct evidence from Chinese populations. This assumption warrants caution and should be evaluated through scenario analyses.
8. The base-case results indicating dominance, with cost savings of $4,045 and an increase of 1.41 QALYs, are significant. However, the negative ICER of -$2,868/QALY is presented without addressing the implications for budget-constrained environments, where initial costs may still present challenges despite potential long-term savings.
9. One-way sensitivity analyses are thorough, highlighting time horizon and psychological costs as significant determinants. The range for PFO closure costs (±50%) may be insufficient due to regional variations in China; an expansion to ±75% could more accurately reflect disparities between urban and rural areas.
10. Probabilistic sensitivity analysis utilizing 10,000 iterations demonstrates methodological rigor, indicating a 94.2% cost-effectiveness at the willingness-to-pay threshold. The description of the scatter plot is informative; however, the manuscript would improve by addressing the 5.8% of iterations in which PFO closure was not cost-effective, along with the parameter combinations that contribute to these outliers.
11. Subgroup analyses based on age and gender provide valuable insights, indicating enhanced benefits in younger patients. A limitation is the lack of subgroups categorized by PFO size or shunt grade, which clinical trials indicate may affect efficacy. Future models should integrate these factors for more accurate recommendations.
12. The budget impact analysis estimates net savings of $68.4 million over a decade, highlighting its relevance to policy considerations. It assumes complete uptake among 4,200 eligible patients each year, neglecting implementation costs such as training or infrastructure, which may lead to an underestimation of short-term burdens.
13. The discussion contextualizes findings within the framework of prior studies; however, it exaggerates the alignment with Wei et al. (ICER $2,783/QALY) as your model demonstrates dominance. It is essential to clarify the differences in assumptions, such as time horizon and costs, to prevent the implication of equivalence.
14. Limitations include the Markov memoryless property and the payer perspective. A further limitation is the omission of procedural complications, such as post-closure atrial fibrillation, beyond mortality, which could influence both costs and utilities if included.
15. The manuscript is well-structured and advocates for the broader adoption of PFO closure in China. To improve scientific rigor, incorporate a CHEERS checklist compliance statement and evaluate multi-way sensitivity analyses for critical parameters such as time horizon and stroke rates.
