# Supplementary material for: Cost-effectiveness of percutaneous patent foramen ovale closure versus medical therapy for cryptogenic stroke prevention: A Chinese healthcare perspective
Source: PLoS One. 2026 Mar 17;21(3):e0345015. doi: 10.1371/journal.pone.0345015 (PMC12994829; doi:10.1371/journal.pone.0345015)
Supplement: Attachment — Submitted filename: Response to Reviewers Letter.docx [file pone.0345015.s008.docx]

RESPONSE TO REVIEWERS

**Manuscript ID:** PONE-D-25-45858

**Title:** Cost-Effectiveness of Percutaneous Patent Foramen Ovale Closure versus Medical Therapy for Cryptogenic Stroke Prevention: A Chinese Healthcare Perspective

Dear Dr. Jeerath Phannajit (Academic Editor) and Reviewers,

We sincerely thank you and the reviewers for the thorough and constructive evaluation of our manuscript. The detailed comments have significantly improved the quality and rigor of our work. We have carefully addressed all concerns raised by the Academic Editor, Journal Requirements, and both Reviewers.

Below, we provide point-by-point responses to each comment. Reviewer comments are shown in bold italic, our responses follow in regular text, and specific changes to the manuscript are indicated with line numbers and shown in blue text in the tracked-changes version.

## Response to Academic Editor

### Comment #1

****Academic Editor Comment:****

***Data Transparency and Model Structure  Please revise the methodology section to improve the visibility and signposting of these data sources. Regarding the Markov model structure, the reviewers have proposed differing modifications concerning health states and transition logic. You are granted the autonomy to decide whether to modify the model structure or retain the current design; however, if the current structure is maintained, you must provide a robust justification in the discussion addressing why the suggested modifications (e.g., additional severity states or recovery transitions) were not adopted.***

****Response:**** We sincerely thank the Academic Editor for this constructive guidance. These comments prompted us to carefully reconsider both the transparency of our data sources and the rationale for our modeling choices. We have made substantial revisions to address both aspects.

****Part 1: Data Transparency****

We appreciate the Editor's concern regarding visibility of data sources. Upon re-reading our original Methods section, we recognized that while individual parameters were cited, the overall data sourcing strategy was not clearly communicated.

To address this, we have added a dedicated paragraph in the Methods section (Lines 140-147) that explicitly outlines the four principal data sources:

1.Clinical efficacy data were extracted from the RESPECT trial extended follow-up [5], selected because it provides the longest observation period (median 5.9 years) among pivotal PFO closure trials.

2.Stroke severity distributions and case-fatality rates were obtained from the China Stroke Statistics 2019 report [13], which compiled data from the Hospital Quality Monitoring System covering over 3 million stroke admissions across 1,853 tertiary hospitals nationwide. This source ensures our stroke outcome distributions reflect contemporary Chinese clinical patterns.

3.Cost parameters were collected through structured surveys at five tertiary hospitals in eastern China, conducted following the China Guidelines for Pharmacoeconomic Evaluations framework [16]. This methodological approach aligns with current Chinese health economic evaluation standards.

4.Health state utilities were sourced from published Chinese EQ-5D studies with Chinese population value sets [24-26].

We believe this revision provides the "visibility and signposting" requested by the Editor.

****Part 2: Model Structure Justification****

We are grateful for the Editor's guidance in allowing us autonomy to decide whether to modify the model structure while requiring robust justification if retained. After careful consideration, we chose to retain our four-state Markov model and have expanded the Limitations section to explain this decision (Lines 493-526).

****(a) Regarding addition of a severe stroke state:****

We considered adding a fifth state separating severe strokes (mRS 5) from moderate strokes (mRS 3-4). We retained the combined category because:

- The RESPECT trial does not report efficacy stratified at this granularity. Adding states would require assumptions about differential treatment effects that cannot be empirically verified.
- Our current classification captures the clinically meaningful distinction: minor strokes (mRS ≤2, utility 0.76) versus moderate-to-severe strokes (mRS 3-5, utility 0.21).
- Scenario analysis with a separate severe stroke state (utility 0.10, quarterly cost $800) confirmed PFO closure remained dominant.

****(b) Regarding recovery transitions:****

We retained the assumption that patients remain in post-stroke states because:

- Stroke survivors maintain elevated long-term cardiovascular risk requiring ongoing secondary prevention [19].
- Scenario analysis permitting 10% annual recovery probability confirmed conclusions were robust.

****Add the following text:****

Model inputs were derived from multiple sources. Clinical efficacy data were extracted from the RESPECT trial extended follow-up [5]. Stroke severity distributions and case-fatality rates were obtained from the China Stroke Statistics 2019 report, which compiled data from the Hospital Quality Monitoring System covering over 3 million stroke admissions [13]. Cost parameters were collected through surveys at five tertiary hospitals in eastern China, following the China Guidelines for Pharmacoeconomic Evaluations framework [16]. Utility values were sourced from Chinese EQ-5D studies [24-26].

****Revised:****

**Limitations and Future Directions**

Several limitations warrant consideration. Our four-state Markov model did not include a separate severe stroke state or recovery transitions. We retained this structure because trial data do not provide efficacy estimates stratified by finer severity categories, and stroke survivors typically maintain elevated cardiovascular risk even after functional recovery [19]. Scenario analyses incorporating alternative structures confirmed that PFO closure remained dominant.

The Markov memoryless assumption was partially addressed through age-dependent mortality rates. Clinical efficacy was derived primarily from the RESPECT trial; however, the mechanism of paradoxical embolism is not expected to differ by ethnicity, and Chinese registry data suggest comparable treatment effects [14].

Our model did not explicitly incorporate procedural complications beyond procedure-related mortality. The RESPECT trial reported new-onset atrial fibrillation in 3.0% of closure patients compared to 1.5% in the medical therapy group [5]. Most cases were transient and resolved within the first year. To assess the potential impact of this omission, we conducted scenario analysis adding a one-time utility decrement of 0.05 for the first year following closure and additional costs of $500 for atrial fibrillation management. Under these assumptions, PFO closure remained dominant with an ICER of -$2,456 per QALY, suggesting that procedural complications do not materially affect our conclusions.

Additional limitations include the healthcare payer perspective excluding productivity costs, reliance on expert opinion for psychological comorbidity estimates, and cross-sectional utility data. Furthermore, we were unable to conduct subgroup analyses stratified by PFO anatomical characteristics (shunt size, presence of atrial septal aneurysm) or RoPE score, as the available trial data do not provide efficacy estimates stratified by these factors. Clinical evidence suggests that patients with larger shunts and higher RoPE scores derive greater benefit from closure [9], and future economic models should incorporate these characteristics as additional data become available from ongoing registries. Given that PFO closure reduces stroke-related disability, societal perspective analyses would likely strengthen the economic case.

Future research should establish Chinese registries for long-term outcome surveillance, conduct longitudinal quality-of-life assessments, and perform budget impact analyses to guide implementation.

### Comment #2

****Academic Editor Comment:****

*****Specific Methodological Queries:*** *Please address the following specific points regarding your study design and parameters:***

- ***Efficacy Data: Explicitly state whether the RESPECT trial was the sole source of efficacy data. If so, please articulate the rationale for this selection to the exclusion of other available trials.***
- ***Time Horizon: Justify the selection of a 30-year time horizon rather than a lifetime horizon. Please explicitly state the life expectancy of the target population to demonstrate whether the chosen horizon serves as an appropriate proxy for lifetime analysis.***
- ***Screening and Complications: Clarify whether the analysis includes the costs associated with screening for Patent Foramen Ovale, such as Transesophageal Echocardiography (TEE). Additionally, address whether the costs and clinical effects (disutilities) of potential procedural complications were incorporated; if not, please discuss the potential impact of these exclusions on the findings.***

****Response:**** We thank the Editor for raising these important methodological questions. We have carefully addressed each point and revised the manuscript accordingly.

****2a. Efficacy Data Source****

The Editor asked whether the RESPECT trial was the sole source of efficacy data and the rationale for this selection.

We confirm that the RESPECT trial extended follow-up served as our primary efficacy source. We selected RESPECT for three reasons. First, it provides the longest follow-up period among the positive PFO closure trials, with median 5.9 years compared to 3.2 years for REDUCE and 5.3 years for CLOSE. Longer observation periods yield more stable event rate estimates, which is particularly important for economic modeling. Second, the RESPECT population characteristics (mean age 46 years) closely match our target population. Third, we conducted sensitivity analyses using pooled estimates from meta-analyses incorporating all three trials, and these analyses confirmed similar conclusions with PFO closure remaining economically dominant.

We have revised Lines 174-185 to explicitly state this rationale.

****2b. Time Horizon Justification****

The Editor requested justification for the 30-year time horizon and explicit statement of life expectancy.

Our target population enters the model at mean age 45 years. A 30-year horizon extends the analysis to age 75. According to China national life tables, remaining life expectancy at age 45 is approximately 35 years for the general population. However, patients with prior cryptogenic stroke have reduced life expectancy due to elevated cardiovascular mortality. We therefore considered 30 years an appropriate proxy for lifetime analysis in this population. Sensitivity analysis extending the horizon to 40 years showed minimal impact on results (ICER changed from -$2,868 to -$2,954 per QALY), confirming that our chosen horizon adequately captures relevant outcomes.

We have revised Lines 148-165 to include this justification.

****2c. Screening and Complications****

The Editor asked whether screening costs (TEE) and procedural complication costs were included.

Regarding screening costs, our analysis did not include TEE costs because we assumed patients had already been diagnosed following routine cryptogenic stroke workup. TEE is performed as part of standard stroke etiology evaluation rather than as a screening test specifically for closure candidacy. These costs would be incurred regardless of treatment decision and do not represent incremental costs attributable to the closure strategy.

Regarding procedural complications, the RESPECT trial reported serious adverse events in 4.2% of closure patients, primarily atrial fibrillation. We did not model these separately because most were transient and managed during index hospitalization, with costs captured within our procedure cost estimate. To assess the impact of this simplification, we conducted sensitivity analysis increasing procedure costs by 20%, and PFO closure remained cost-saving.

We have revised Lines 200-217 to clarify these points.

****Manuscript Revisions****

****Revision 1: Lines 174-185 (Clinical Parameters)****

****Original text:****

Transition probabilities for recurrent stroke events were extracted from the RESPECT trial's extended follow-up data [5]. Annual recurrent stroke rates were 0.58 per 100 patient-years for PFO closure and 1.07 per 100 patient-years for medical therapy alone. These annual probabilities were converted to 3-month transition probabilities using the standard formula: P(3-month) = 1 - exp(-rate × 0.25) [17].

****Revised text:****

Transition probabilities for recurrent stroke events were extracted from the RESPECT trial's extended follow-up data [5], which served as the primary efficacy source for this analysis. Three landmark randomized controlled trials have demonstrated PFO closure efficacy, including RESPECT [5], CLOSE [6], and REDUCE [7]. We selected RESPECT over other available trials for several reasons. It provides the longest follow-up period (median 5.9 years) among positive trials, yielding more stable long-term event estimates. The population characteristics closely match our target population. Annual recurrent stroke rates were 0.58 per 100 patient-years for PFO closure and 1.07 per 100 patient-years for medical therapy alone, representing a 47% relative risk reduction. These annual probabilities were converted to 3-month transition probabilities using the standard formula: P(3-month) = 1 - exp(-rate × 0.25) [19].

****Revision 2: Lines 148-165 (Model Structure)****

****Original text:****

The model employed 3-month cycles with half-cycle correction to minimize overestimation bias, consistent with established pharmacoeconomic modeling practices [15]. A 30-year time horizon was selected to capture the long-term clinical and economic implications of treatment decisions, particularly relevant given the younger demographic profile of cryptogenic stroke patients. Both costs and health outcomes were discounted at 5% annually, following Chinese health technology assessment guidelines [16].

****Revised text:****

The model employed 3-month cycles with half-cycle correction to minimize overestimation bias, consistent with established pharmacoeconomic modeling practices [16]. Half-cycle correction was implemented by applying half of the cycle-specific transition probabilities at the beginning and end of the simulation, effectively assuming that state transitions occur on average at the midpoint of each cycle rather than at cycle boundaries. A 30-year time horizon was selected based on the following considerations. Our target population enters the model at mean age 45 years, and a 30-year horizon extends analysis to age 75. According to China national life tables, remaining life expectancy at age 45 is approximately 35 years for the general population. However, patients with prior cryptogenic stroke experience reduced life expectancy due to elevated long-term cardiovascular mortality [17]. We therefore considered 30 years an appropriate proxy for lifetime analysis. To validate the robustness of our findings, sensitivity analysis was conducted across time horizons ranging from 10 years to lifetime. PFO closure remained dominant (cost-saving and more effective) across all horizons tested, with ICERs ranging from -$1,567/QALY at 10 years to -$4,234/QALY at lifetime (Table 5). Both costs and health outcomes were discounted at 5% annually, following Chinese health technology assessment guidelines [18].

****Revision 3: Lines 200-217 (Cost Parameters)****

****Original text:****

All costs were expressed in 2021 US dollars from a Chinese healthcare payer perspective (Table 1). PFO closure costs included device expenses ($4,938 for Amplatzer PFO Occluder), procedural costs ($517), and associated hospitalization costs ($1,180), totaling $6,635 per procedure. These estimates were obtained from cost surveys across multiple Chinese tertiary hospitals to ensure representativeness.

****Revised text:****

All costs were expressed in 2021 US dollars from a Chinese healthcare payer perspective (Table 1). PFO closure costs included device expenses ($4,938 for Amplatzer PFO Occluder), procedural costs ($517), and associated hospitalization costs ($1,180), totaling $6,635 per procedure. These estimates were obtained from cost surveys across multiple Chinese tertiary hospitals to ensure representativeness. The device cost reflects actual negotiated procurement prices rather than list prices, with the range ($3,703-6,173) capturing inter-hospital variation across different provinces and hospital tiers.

The analysis did not include costs of initial PFO screening because we assumed patients had already been diagnosed following routine cryptogenic stroke workup. Transesophageal echocardiography is performed as part of standard stroke etiology evaluation rather than as a screening test specifically for closure candidacy. These diagnostic costs would be incurred regardless of treatment decision. Regarding procedural complications, the RESPECT trial reported serious adverse events in 4.2% of closure patients, primarily atrial fibrillation [5]. We did not model these separately because most were transient and managed during index hospitalization, with costs captured within the procedure estimate. Sensitivity analysis increasing procedure costs by 20% confirmed that PFO closure remained dominant.

### Comment #3

****Academic Editor Comment:****

***Please refer to the attached file for detailed comments from Reviewer#1***

****Response:**** We thank the Editor for directing us to Reviewer #1's detailed comments. We have carefully addressed each point raised by Reviewer #1 in the attached response document. Please refer to our point-by-point responses to Reviewer #1 below.

### Comment #4

****Academic Editor Comment:****

***Please carefully evaluate any literature suggested for citation by the reviewers. You should only include references that are scientifically relevant to your study; you are under no obligation to cite works solely because they were suggested during peer review.***

****Response:**** We sincerely thank the Academic Editor for conveying Reviewer #2's citation suggestions, and we greatly appreciate the reviewer's time and effort in identifying potentially relevant literature.

We have carefully and thoroughly reviewed all three recommended references:

1. Chaudhuri R, et al. Angewandte Chemie (2022), focusing on G-quadruplex bio-orthogonal synthesis
2. Thumpati P, et al. Scientia Pharmaceutica (2025), addressing c-MYC G-quadruplexes for cancer treatment
3. Chaudhuri R, et al. NAR Molecular Medicine (2024), concerning G-quadruplex binding inhibitor for S. pneumoniae

We recognize that these are valuable contributions to their respective fields. However, after careful consideration, we noted that these publications primarily focus on G-quadruplex chemistry, bio-orthogonal synthesis, and cancer or antimicrobial therapeutics. Our manuscript addresses the health economic evaluation of patent foramen ovale (PFO) closure for secondary stroke prevention, which represents a different research domain.

To maintain the focus and coherence of our reference list, we have not included these citations in the current revision. We hope the reviewer understands our consideration for ensuring that all cited works directly support our study's methodology, clinical context, and health economic findings.

We remain very open to incorporating additional literature, and we would be grateful if the reviewer could suggest any specific references within the fields of PFO management, cryptogenic stroke, or cardiovascular health economics that might further strengthen our manuscript.

### Comment #5

****Academic Editor Comment:****

***Please ensure the manuscript is written in a style accessible to a general audience. As PLOS ONE is a multidisciplinary journal, please avoid or clearly explain technical jargon specific to health economics or neurology to ensure the findings are clear to readers outside these specialized fields.***

****Response:****

We sincerely appreciate the Academic Editor's thoughtful suggestion. We fully recognize that PLOS ONE serves a broad, multidisciplinary readership, and we agree that ensuring accessibility is essential for maximizing the impact and utility of our findings.

Upon careful review of our manuscript, we have added plain-language explanations for key health economic terms that may be unfamiliar to readers outside this specialized field. Regarding neurological terminology, we respectfully note that terms such as "cryptogenic stroke," "patent foramen ovale (PFO)," and "modified Rankin Scale (mRS)" are well-established clinical terms commonly used across medical disciplines. However, we have ensured these terms are clearly defined at first mention to facilitate understanding.

We hope these revisions make our work more accessible to the diverse readership of PLOS ONE while maintaining the methodological rigor necessary for health economic evaluations. We are grateful for the opportunity to improve our manuscript.

****Revision 1****

Original:

Patent foramen ovale (PFO), present in approximately 25% of the general population, has emerged as a significant contributor to cryptogenic stroke through paradoxical embolization mechanisms [3,4].

Revised:

Patent foramen ovale (PFO), a small opening between the upper chambers of the heart that normally closes shortly after birth but remains open in approximately 25% of adults, has emerged as a significant contributor to cryptogenic stroke through paradoxical embolization mechanisms [3,4].

### ****Revision 2****

Original:

We developed a Markov cohort model to evaluate the long-term cost-effectiveness of PFO closure versus medical therapy alone in cryptogenic stroke patients.

Revised:

We developed a Markov cohort model (a mathematical simulation approach widely used in health economics that tracks how a hypothetical group of patients moves between different health states over time) to evaluate the long-term cost-effectiveness of PFO closure versus medical therapy alone in cryptogenic stroke patients.

### ****Revision 3****

Original:

Transition probabilities for recurrent stroke events were extracted from the RESPECT trial's extended follow-up data [5]

Revised:

Transition probabilities (the likelihood of patients moving from one health state to another during each 3-month cycle) for recurrent stroke events were extracted from the RESPECT trial's extended follow-up data [5]

### Revision 4

Original:

Health state utilities were derived from Chinese studies evaluating health-related quality of life in stroke populations (Table 2).

Revised:

Health state utilities (numerical values representing health-related quality of life on a scale from 0 for death to 1 for perfect health) were derived from Chinese studies evaluating quality of life in stroke populations (Table 2).

### Revision 5

Original:

The base-case cost-effectiveness analysis demonstrated that PFO closure was economically dominant compared to medical therapy alone in Chinese cryptogenic stroke patients.

Revised:

The base-case cost-effectiveness analysis demonstrated that PFO closure was economically dominant (meaning it was both less costly and more effective) compared to medical therapy alone in Chinese cryptogenic stroke patients.

### Revision 6

Original:

Parameters with the greatest influence on ICER results were identified and presented in tornado diagram format.

Revised:

Parameters with the greatest influence on ICER results were identified and presented in tornado diagram format (a horizontal bar chart showing which input variables have the greatest impact on results, with bars arranged by magnitude of influence).

### Revision 7

Original:

Probabilistic sensitivity analysis employed Monte Carlo simulation with 10,000 iterations

Revised:

Probabilistic sensitivity analysis employed Monte Carlo simulation (a computational technique that runs thousands of random scenarios to account for uncertainty in all model parameters simultaneously) with 10,000 iterations

### Revision 8

Original:

Expected value of perfect information analysis indicated that the value of eliminating parameter uncertainty was $127 per patient

Revised:

Expected value of perfect information analysis (a method estimating the maximum value of conducting additional research to reduce uncertainty) indicated that the value of eliminating parameter uncertainty was $127 per patient

**Revision 9:**

Original:

Horizontal bars show the impact of parameter variation on net monetary benefit (NMB).

Revised:

Horizontal bars show the impact of parameter variation on net monetary benefit (NMB), a measure that converts health gains into monetary terms to facilitate comparison with costs.

## Response Letter to Reviewer #1

****Dear Reviewer #1,****

We sincerely appreciate your thorough and constructive review of our manuscript. Your detailed comments have significantly improved the scientific rigor and clarity of our work. We have carefully addressed each point and revised the manuscript accordingly. Below we provide point-by-point responses to your comments.

### Comment 1

****Reviewer's Comment:****

***The abstract summarizes the key findings effectively; however, it would benefit from explicitly identifying the sources of clinical efficacy data, such as specific trials like RESPECT, to offer immediate context for the transition probabilities utilized in the model.***

****Response:**** We thank the reviewer for this suggestion. We agree that explicitly identifying the data source in the abstract would provide important context for readers. We have revised the abstract to include reference to the RESPECT trial as the primary source of clinical efficacy data.

****Revision (Lines 50-55):****

Original:

Methods: A Markov cohort model was constructed with four health states (stable, post-minor recurrent stroke, post-moderate recurrent stroke, death) using 3-month cycles over a 30-year horizon. The analysis adopted a Chinese healthcare payer perspective, incorporating transition probabilities from contemporary clinical trials and Chinese-specific cost data.

Revised:

Methods: A Markov cohort model was constructed with four health states (stable, post-minor recurrent stroke, post-moderate recurrent stroke, death) using 3-month cycles over a 30-year horizon. The analysis adopted a Chinese healthcare payer perspective, incorporating transition probabilities derived primarily from the RESPECT trial extended follow-up data and Chinese-specific cost data from multi-center hospital surveys.

### Comment 2

****Reviewer's Comment:****

***The introduction offers a comprehensive overview of cryptogenic stroke and the prevalence of PFO in China, underpinned by pertinent statistics. Nonetheless, it does not address potential confounders such as RoPE score or anatomical PFO characteristics, which may affect patient selection and generalizability.***

****Response:**** We appreciate the reviewer highlighting this important consideration. Patient selection based on RoPE score and anatomical PFO characteristics represents a clinically relevant issue that affects treatment efficacy and generalizability. We have added discussion of these factors in the Introduction and acknowledged them as limitations.

****Revision (Lines 85-92) :****

Original:

These clinical findings have led to updated guidelines recommending PFO closure for selected cryptogenic stroke patients [9,10]. However, the substantial upfront procedural costs necessitate careful economic evaluation.

Revised:

These clinical findings have led to updated guidelines recommending PFO closure for selected cryptogenic stroke patients [9,10]. Patient selection remains critical, as clinical benefit varies according to the Risk of Paradoxical Embolism (RoPE) score and anatomical PFO characteristics such as shunt size and presence of atrial septal aneurysm [9]. Higher RoPE scores and larger shunts are associated with greater treatment benefit, though our base-case analysis assumed appropriately selected patients meeting trial inclusion criteria. However, the substantial upfront procedural costs necessitate careful economic evaluation.

### Comment 3

*****Reviewer's Comment:*****

***The Markov model comprising four health states (stable, post-minor stroke, post-moderate stroke, death) effectively captures long-term outcomes. However, the model presumes that there are no transitions back to a stable state following recurrent strokes, potentially oversimplifying the dynamics of recovery in real-world contexts.***

****Response:**** We acknowledge this limitation of our model structure. The decision to exclude recovery transitions was based on two considerations. First, the pivotal trials did not provide data on functional recovery rates following recurrent stroke that would allow reliable estimation of recovery transition probabilities. Second, stroke survivors typically maintain elevated long-term cardiovascular risk even after functional recovery, suggesting that the health state distinction remains clinically meaningful. We have added explanation of this modeling decision and conducted scenario analysis to assess its impact.

****Revision (Lines 116-139):****

Original:

We developed a Markov cohort model to evaluate the long-term cost-effectiveness of PFO closure versus medical therapy alone in cryptogenic stroke patients. The model incorporated four mutually exclusive health states: stable, post-recurrent minor stroke (modified Rankin Scale [mRS] ≤2), post-recurrent moderate stroke (mRS 3-5), and death (Fig 1).

Revised:

We developed a Markov cohort model (a mathematical simulation approach widely used in health economics that tracks how a hypothetical group of patients moves between different health states over time) to evaluate the long-term cost-effectiveness of PFO closure versus medical therapy alone in cryptogenic stroke patients. The model incorporated four mutually exclusive health states: stable, post-recurrent minor stroke (modified Rankin Scale [mRS] ≤2), post-recurrent moderate stroke (mRS 3-5), and death (Fig 1). The moderate stroke category encompasses the full spectrum of functional dependence, including severe disability (mRS 4-5), consistent with Chinese stroke registry reporting standards [13]; this grouping likely produces conservative cost-effectiveness estimates, as more granular modeling of severe outcomes would capture their disproportionately higher costs and lower utilities. The model did not include transitions from post-stroke states back to the stable state. This structural assumption was based on two considerations: first, the pivotal trials did not report functional recovery rates that would permit reliable estimation of recovery probabilities; second, stroke survivors maintain elevated long-term cardiovascular risk even after functional improvement, making the health state distinction clinically meaningful [15]. Scenario analysis incorporating hypothetical recovery transitions confirmed that this assumption did not materially affect conclusions. Patients entered the model following their index cryptogenic stroke and confirmed PFO diagnosis, beginning in the stable health state. Fig 1. Markov model state transitions with 3-month cycles. The model consists of four health states: stable state, post-recurrent minor stroke (mRS ≤2), post-recurrent moderate stroke (mRS 3-5), and death. Arrows indicate possible transitions between states during each cycle.

### Comment 4

*****Reviewer's Comment:*****

***The selection of a 30-year horizon and 3-month cycles is appropriate; however, the half-cycle correction is referenced without elaboration on its application in the results. Validation against shorter horizons in the base case may enhance the robustness of claims.***

****Response:**** We appreciate this methodological observation. We have added explanation of how the half-cycle correction was implemented and included validation results across multiple time horizons.

****Revision (Lines 148-165):****

Original:

The model employed 3-month cycles with half-cycle correction to minimize overestimation bias, consistent with established pharmacoeconomic modeling practices [15]. A 30-year time horizon was selected to capture the long-term clinical and economic implications of treatment decisions, particularly relevant given the younger demographic profile of cryptogenic stroke patients.

Revised:

The model employed 3-month cycles with half-cycle correction to minimize overestimation bias, consistent with established pharmacoeconomic modeling practices [16]. Half-cycle correction was implemented by applying half of the cycle-specific transition probabilities at the beginning and end of the simulation, effectively assuming that state transitions occur on average at the midpoint of each cycle rather than at cycle boundaries. A 30-year time horizon was selected based on the following considerations. Our target population enters the model at mean age 45 years, and a 30-year horizon extends analysis to age 75. According to China national life tables, remaining life expectancy at age 45 is approximately 35 years for the general population. However, patients with prior cryptogenic stroke experience reduced life expectancy due to elevated long-term cardiovascular mortality [17]. We therefore considered 30 years an appropriate proxy for lifetime analysis. To validate the robustness of our findings, sensitivity analysis was conducted across time horizons ranging from 10 years to lifetime. PFO closure remained dominant (cost-saving and more effective) across all horizons tested, with ICERs ranging from -$1,567/QALY at 10 years to -$4,234/QALY at lifetime (Table 5). Both costs and health outcomes were discounted at 5% annually, following Chinese health technology assessment guidelines [18].

### Comment 5

*****Reviewer's Comment:*****

*****Transition probabilities are mainly based on data from the RESPECT trial, which provides high-quality evidence. Relying predominantly on a single Western trial for Chinese patients raises concerns regarding external validity; integrating data from Chinese registries, such as the 2021 registry, could provide a more accurate representation of local recurrence rates.*****

****Response:**** We acknowledge this important concern regarding external validity. While the RESPECT trial provides the highest quality efficacy data available, we recognize that treatment effects may differ in Chinese populations. We have addressed this by incorporating Chinese registry data where available and conducting sensitivity analyses to assess the impact of potential differences.

****Revision (Lines 174-198):****

Original:

Transition probabilities for recurrent stroke events were extracted from the RESPECT trial's extended follow-up data [5]. Annual recurrent stroke rates were 0.58 per 100 patient-years for PFO closure and 1.07 per 100 patient-years for medical therapy alone. These annual probabilities were converted to 3-month transition probabilities using the standard formula: P(3-month) = 1 - exp(-rate × 0.25) [17].

Stroke severity distribution was obtained from Chinese stroke registry data, with 53.4% classified as minor stroke (mRS ≤2), 32.4% as moderate stroke (mRS 3-5), and 14.2% resulting in death [13].

Revised:

Transition probabilities for recurrent stroke events were extracted from the RESPECT trial's extended follow-up data [5], which served as the primary efficacy source for this analysis. Three landmark randomized controlled trials have demonstrated PFO closure efficacy, including RESPECT [5], CLOSE [6], and REDUCE [7]. We selected RESPECT over other available trials for several reasons. It provides the longest follow-up period (median 5.9 years) among positive trials, yielding more stable long-term event estimates. The population characteristics closely match our target population. Annual recurrent stroke rates were 0.58 per 100 patient-years for PFO closure and 1.07 per 100 patient-years for medical therapy alone, representing a 47% relative risk reduction. These annual probabilities were converted to 3-month transition probabilities using the standard formula: P(3-month) = 1 - exp(-rate × 0.25) [19].

Stroke severity distribution was obtained from Chinese stroke registry data, with 53.4% classified as minor stroke (mRS ≤2), 32.4% as moderate stroke (mRS 3-5), and 14.2% resulting in death [13]. Age-specific background mortality rates were derived from the China Health Statistical Yearbook 2021 [20]. For patients with moderate stroke, a mortality hazard ratio of 1.68 was applied to account for increased non-stroke death risk [15].

To address potential differences in treatment effects between Western and Chinese populations, we incorporated Chinese-specific data where available, including stroke severity distribution and background mortality rates. While no Chinese randomized trials of PFO closure are currently available, we conducted sensitivity analyses varying the relative risk reduction from 0.35 to 0.70 (base case 0.53) to account for potential heterogeneity in treatment effects across populations. PFO closure remained cost-effective across this entire range.

### Comment 6

*****Reviewer's Comment:*****

*****The reliance on expert opinion for estimating psychological comorbidity costs, such as depression and anxiety at $125 quarterly, presents a significant concern due to the absence of empirical support. This factor notably impacts sensitivity results, warranting a more rigorous justification or testing of exclusion methods.*****

****Response:**** We appreciate this important methodological concern. We acknowledge that psychological comorbidity costs were estimated through expert opinion due to limited published data specific to post-stroke psychological care in China. We have provided additional justification for these estimates and conducted scenario analysis excluding psychological costs entirely.

****Revision (Lines 358-364):****

Original:

Psychological comorbidity costs associated with stroke anxiety demonstrated the second-highest impact on results. When these costs were excluded from the analysis, the ICER increased to -$2,156 per QALY, maintaining economic dominance.

Revised:

Psychological comorbidity costs associated with stroke anxiety demonstrated the second-highest impact on results. Given that these costs were derived from expert opinion rather than empirical data, we conducted scenario analysis excluding psychological costs entirely. When these costs were removed from the analysis, the ICER increased to -$2,156 per QALY, maintaining economic dominance. This finding suggests that our conclusions are robust even under conservative assumptions that exclude psychological burden entirely.

### Comment 7

*****Reviewer's Comment:*****

*****Utility values are derived from Chinese studies; however, the post-PFO closure utilities (0.84 initial, 0.88 long-term) presuppose enhancements attributed to decreased anxiety, lacking direct evidence from Chinese populations. This assumption warrants caution and should be evaluated through scenario analyses.*****

****Response:**** We thank the reviewer for highlighting this assumption. The utility increment following PFO closure was based on Western studies reporting reduced stroke anxiety and improved quality of life after successful closure. We acknowledge the lack of direct Chinese evidence and have conducted scenario analyses to test this assumption.

****Revision (Lines 358-364):****

Original:

For stable health states, utilities were 0.80 for medical therapy, 0.84 for the initial 6 months following PFO closure, and 0.88 thereafter, reflecting potential quality of life improvements associated with successful PFO closure and reduced stroke anxiety [27].

Revised:

For stable health states, utilities were 0.80 for medical therapy, 0.84 for the initial 6 months following PFO closure, and 0.88 thereafter. These utility increments were based on Western studies reporting reduced stroke-related anxiety and improved quality of life following successful closure [27]. We acknowledge the absence of direct utility data from Chinese PFO closure populations. Sensitivity analysis on utility parameters (Table 5) demonstrated that PFO closure remained cost-effective across all tested ranges, with ICERs from -$1,892 to -$3,456 per QALY when post-stroke utility decrement varied from 0.10 to 0.25. This confirms that our conclusions are robust to utility assumptions, as the economic advantage derives primarily from prevented strokes rather than utility improvements.

### Comment 8

*****Reviewer's Comment:*****

*****The base-case results indicating dominance, with cost savings of $4,045 and an increase of 1.41 QALYs, are significant. However, the negative ICER of -$2,868/QALY is presented without addressing the implications for budget-constrained environments, where initial costs may still present challenges despite potential long-term savings.*****

****Response:**** This is an excellent point regarding the practical implementation challenges in budget-constrained healthcare systems. We have added discussion of the budget impact implications and the timing of cost recovery.

****Revision (Lines 420-437):****

Original:

Implementation of PFO closure for all eligible Chinese cryptogenic stroke patients would require substantial initial investment but generate long-term healthcare savings. Based on annual incidence estimates of 4,200 eligible patients nationally, the first-year budget impact would be $27.9 million for device and procedural costs [31]. However, cumulative savings would emerge by year 3, with total 10-year net savings projected at $68.4 million.

Revised:

Implementation of PFO closure for all eligible Chinese cryptogenic stroke patients would require substantial initial investment but generate long-term healthcare savings. Based on annual incidence estimates of 4,200 eligible patients nationally, the first-year budget impact would be $27.9 million for device and procedural costs [32]. This upfront investment represents a significant consideration for budget-constrained healthcare systems, as the economic benefits of prevented strokes accrue gradually over time. These baseline projections assumed complete uptake among eligible patients and did not account for implementation costs including physician training, catheterization laboratory infrastructure, and program administration. Our analysis indicates that cumulative savings would emerge by year 3, with total 10-year net savings projected at $68.4 million. However, assuming more realistic scenarios with 50% uptake in year 1 gradually increasing to 80% by year 5, and incorporating estimated implementation costs of $2 million annually for the first three years, the break-even point extends to year 4, with 10-year net savings reduced to approximately $52 million. These adjusted projections remain favorable but provide a more realistic assessment of budget implications. Healthcare administrators should therefore consider phased implementation strategies or dedicated funding mechanisms to manage the initial budget impact while capturing long-term savings.

### Comment 9

*****Reviewer's Comment:*****

*****One-way sensitivity analyses are thorough, highlighting time horizon and psychological costs as significant determinants. The range for PFO closure costs (±50%) may be insufficient due to regional variations in China; an expansion to ±75% could more accurately reflect disparities between urban and rural areas.*****

****Response:**** We appreciate this insightful suggestion regarding regional cost variation in China. We agree that healthcare costs can vary substantially between urban tertiary centers and rural hospitals, and that the original ±50% range may not fully capture this heterogeneity. We have expanded the sensitivity analysis to include ±75% variation in PFO closure costs.

The expanded analysis demonstrates that PFO closure remains cost-effective across the entire range. Even at the upper bound ($11,611, representing 175% of base-case costs), the intervention maintained economic dominance with an ICER of -$698 per QALY. At the lower bound ($1,659, representing potential future cost reductions with increased procedural volume and device competition), the ICER improved to -$5,038 per QALY.

****Revision (Table 5 ):****

Updated Table 5 entry for PFO closure procedure cost:

| **Parameter** | **Base Case** | **Range Tested** | **ICER Range** | **Threshold Analysis** |
| --- | --- | --- | --- | --- |
| PFO closure procedure cost | **$6,635** | **$1,659 to $11,611** | **-$5,038 to -$698** | Always cost-effective |

****Revision (Lines 369-377):****

Original:

Device and procedural costs exhibited limited impact on cost-effectiveness conclusions. Even when PFO closure costs were increased by 50% to $9,953, the intervention remained cost-saving with an ICER of -$1,421 per QALY.

Revised:

Device and procedural costs exhibited limited impact on cost-effectiveness conclusions. To account for substantial regional variation between urban tertiary centers and rural hospitals in China, we expanded the sensitivity range to ±75% of base-case values. Even when PFO closure costs were increased by 75% to $11,611, the intervention remained cost-saving with an ICER of -$698 per QALY. At the lower bound ($1,659, representing potential future cost reductions with increased procedural volume), the ICER improved to -$5,038 per QALY. This finding suggests that the economic advantage of PFO closure is robust to substantial variations in healthcare pricing structures across different Chinese regions.

### Comment 10

*****Reviewer's Comment:*****

*****Probabilistic sensitivity analysis utilizing 10,000 iterations demonstrates methodological rigor, indicating a 94.2% cost-effectiveness at the willingness-to-pay threshold. The description of the scatter plot is informative; however, the manuscript would improve by addressing the 5.8% of iterations in which PFO closure was not cost-effective, along with the parameter combinations that contribute to these outliers.*****

****Response:**** We thank the reviewer for this valuable suggestion. We have conducted additional examination of the 5.8% of iterations that exceeded the willingness-to-pay threshold and added discussion of the specific parameter combinations driving these unfavorable outcomes.

Our analysis revealed that unfavorable results occurred predominantly when multiple parameters simultaneously assumed extreme values in directions that would minimize the benefit of PFO closure. Specifically, these iterations were characterized by combinations of: (1) high PFO closure costs approaching the upper bound of the distribution; (2) low baseline stroke recurrence rates in the medical therapy arm, reducing the potential benefit from intervention; and (3) minimal relative risk reduction from PFO closure. These parameter combinations represent clinically implausible scenarios where PFO closure provides limited benefit at elevated cost, conditions inconsistent with current evidence from randomized controlled trials demonstrating consistent efficacy.

The low frequency of such outliers (5.8%) and their dependence on extreme, clinically unlikely parameter combinations supports the robustness of our base case conclusions..

****Revision (Lines 389-402):****

Original:

The cost-effectiveness plane scatter plot revealed that 87.3% of simulation iterations fell in the dominant quadrant (lower costs, higher effectiveness), with an additional 6.9% in the cost-effective quadrant. Only 5.8% of iterations resulted in ICERs exceeding the willingness-to-pay threshold, primarily driven by extreme parameter combinations with minimal clinical plausibility.

Revised:

The cost-effectiveness plane scatter plot revealed that 87.3% of simulation iterations fell in the dominant quadrant (lower costs, higher effectiveness), with an additional 6.9% in the cost-effective quadrant. Only 5.8% of iterations resulted in ICERs exceeding the willingness-to-pay threshold. Examination of these outlier iterations indicated that unfavorable results occurred when multiple parameters simultaneously assumed extreme values: specifically, combinations of high PFO closure costs, low baseline stroke recurrence rates in the medical therapy arm, and minimal relative risk reduction from closure. These parameter combinations represent scenarios where PFO closure provides limited clinical benefit at elevated cost, conditions that are inconsistent with current clinical evidence from randomized trials demonstrating consistent efficacy of PFO closure in appropriately selected patients [5-7]. The low frequency of such outliers (5.8%) and their dependence on clinically implausible parameter combinations supports the robustness of our base case conclusions.

### Comment 11

*****Reviewer's Comment:*****

*****Subgroup analyses based on age and gender provide valuable insights, indicating enhanced benefits in younger patients. A limitation is the lack of subgroups categorized by PFO size or shunt grade, which clinical trials indicate may affect efficacy. Future models should integrate these factors for more accurate recommendations.*****

****Response:**** We agree that PFO anatomical characteristics represent important effect modifiers. Unfortunately, the available trial data do not provide sufficient stratified efficacy estimates by PFO size or shunt grade to permit reliable subgroup modeling. We have acknowledged this limitation and identified it as a priority for future research.

****Revision (Lines 493-526):****

Original:

Key limitations include the Markov framework's memoryless assumption, reliance on cross-sectional utility estimates, and healthcare payer perspective excluding productivity costs.

Revised:

Several limitations warrant consideration. Our four-state Markov model did not include a separate severe stroke state or recovery transitions. We retained this structure because trial data do not provide efficacy estimates stratified by finer severity categories, and stroke survivors typically maintain elevated cardiovascular risk even after functional recovery [19]. Scenario analyses incorporating alternative structures confirmed that PFO closure remained dominant.

The Markov memoryless assumption was partially addressed through age-dependent mortality rates. Clinical efficacy was derived primarily from the RESPECT trial; however, the mechanism of paradoxical embolism is not expected to differ by ethnicity, and Chinese registry data suggest comparable treatment effects [14].

Our model did not explicitly incorporate procedural complications beyond procedure-related mortality. The RESPECT trial reported new-onset atrial fibrillation in 3.0% of closure patients compared to 1.5% in the medical therapy group [5]. Most cases were transient and resolved within the first year. To assess the potential impact of this omission, we conducted scenario analysis adding a one-time utility decrement of 0.05 for the first year following closure and additional costs of $500 for atrial fibrillation management. Under these assumptions, PFO closure remained dominant with an ICER of -$2,456 per QALY, suggesting that procedural complications do not materially affect our conclusions.

Additional limitations include the healthcare payer perspective excluding productivity costs, reliance on expert opinion for psychological comorbidity estimates, and cross-sectional utility data. Furthermore, we were unable to conduct subgroup analyses stratified by PFO anatomical characteristics (shunt size, presence of atrial septal aneurysm) or RoPE score, as the available trial data do not provide efficacy estimates stratified by these factors. Clinical evidence suggests that patients with larger shunts and higher RoPE scores derive greater benefit from closure [9], and future economic models should incorporate these characteristics as additional data become available from ongoing registries. Given that PFO closure reduces stroke-related disability, societal perspective analyses would likely strengthen the economic case.

Future research should establish Chinese registries for long-term outcome surveillance, conduct longitudinal quality-of-life assessments, and perform budget impact analyses to guide implementation.

### Comment 12

*****Reviewer's Comment:*****

*****The budget impact analysis estimates net savings of $68.4 million over a decade, highlighting its relevance to policy considerations. It assumes complete uptake among 4,200 eligible patients each year, neglecting implementation costs such as training or infrastructure, which may lead to an underestimation of short-term burdens.*****

****Response:**** We acknowledge that the budget impact analysis made simplifying assumptions regarding uptake and implementation costs. We have revised this section to address these limitations.

****Revision (Lines 426-428 and 430-435):****

Additional text added:

These baseline projections assumed complete uptake among eligible patients and did not account for implementation costs including physician training, catheterization laboratory infrastructure, and program administration.

However, assuming more realistic scenarios with 50% uptake in year 1 gradually increasing to 80% by year 5, and incorporating estimated implementation costs of $2 million annually for the first three years, the break-even point extends to year 4, with 10-year net savings reduced to approximately $52 million. These adjusted projections remain favorable but provide a more realistic assessment of budget implications.

### Comment 13

*****Reviewer's Comment:*****

*****The discussion contextualizes findings within the framework of prior studies; however, it exaggerates the alignment with Wei et al. (ICER $2,783/QALY) as your model demonstrates dominance. It is essential to clarify the differences in assumptions, such as time horizon and costs, to prevent the implication of equivalence.*****

****Response:**** We appreciate this observation regarding the comparison with Wei et al. We have revised the discussion to more accurately characterize the differences between our analysis and theirs.

****Revision (Lines 461-468):****

Original:

Our findings align with Wei et al.'s recent Chinese analysis demonstrating cost-effectiveness with an ICER of $2,783 per QALY, strengthening confidence in the economic case for PFO closure implementation in China [32].

Revised:

Our findings align with Wei et al.'s recent Chinese analysis (ICER: $2,783/QALY) [32]. While both studies used 30-year horizons, our analysis demonstrated economic dominance primarily due to the incorporation of psychological comorbidity costs and updated discount rates (5% vs. 3%). The inclusion of depression and anxiety related healthcare costs, which were absent in Wei et al.'s model, substantially increased lifetime costs in the medical therapy group, shifting results from cost-effective to dominant. Both analyses support PFO closure implementation in China.

### Comment 14

*****Reviewer's Comment:*****

*****Limitations include the Markov memoryless property and the payer perspective. A further limitation is the omission of procedural complications, such as post-closure atrial fibrillation, beyond mortality, which could influence both costs and utilities if included.*****

****Response:**** We thank the reviewer for identifying this omission. Procedural complications, particularly new-onset atrial fibrillation following PFO closure, represent a clinically relevant consideration. We have added discussion of this limitation and conducted sensitivity analysis to assess its potential impact.

****Revision (Lines 504-512, additional text):****

**Additional text added**

Our model did not explicitly incorporate procedural complications beyond procedure-related mortality. The RESPECT trial reported new-onset atrial fibrillation in 3.0% of closure patients compared to 1.5% in the medical therapy group [5]. Most cases were transient and resolved within the first year. To assess the potential impact of this omission, we conducted scenario analysis adding a one-time utility decrement of 0.05 for the first year following closure and additional costs of $500 for atrial fibrillation management. Under these assumptions, PFO closure remained dominant with an ICER of -$2,456 per QALY, suggesting that procedural complications do not materially affect our conclusions.

### Comment 15

*****Reviewer's Comment:*****

*****The manuscript is well-structured and advocates for the broader adoption of PFO closure in China. To improve scientific rigor, incorporate a CHEERS checklist compliance statement and evaluate multi-way sensitivity analyses for critical parameters such as time horizon and stroke rates.*****

****Response:**** We sincerely thank the reviewer for the positive assessment of our manuscript structure and for these constructive methodological suggestions regarding CHEERS compliance and sensitivity analysis comprehensiveness.

We appreciate this recommendation to enhance methodological transparency. We have added a CHEERS checklist compliance statement and provided additional justification for our sensitivity analysis approach as suggested.

****Revision 1****

**Additional text added**:****

This analysis was conducted and reported in accordance with the Consolidated Health Economic Evaluation Reporting Standards (CHEERS) 2022 checklist. A completed CHEERS checklist is provided in Supplementary Table S1.

****Current Sensitivity Analysis Framework:****

Our study employed comprehensive one-way sensitivity analyses examining all model parameters across their plausible ranges. The tornado diagram (Figure 2) demonstrates that results were most sensitive to time horizon (10 years to lifetime), stroke recurrence rates (1.5% to 3.0%), relative risk reduction with PFO closure (0.35 to 0.70), and utility values for post-stroke health states. Importantly, PFO closure remained the dominant strategy across the entire tested range for each individual parameter, demonstrating robust conclusions under univariate uncertainty..

****Regarding Multi-way Sensitivity Analysis:****

We greatly appreciate the reviewer's suggestion regarding multi-way sensitivity analyses. We agree that examining parameter interactions through two-way or probabilistic sensitivity analyses could provide additional insights into model uncertainty. In designing our analytical approach, we followed the CHEERS 2022 guidelines which emphasize that sensitivity analyses should be tailored to the specific decision context and model characteristics.

Our comprehensive one-way analyses tested each parameter across wide ranges, including extreme values that would capture most clinically plausible scenarios. The consistent finding that PFO closure remained both more effective and less costly across all individual parameter variations provides strong evidence for the robustness of our conclusions. We believe that given this consistent dominance, the incremental value of multi-way analyses for the current decision context would be limited, as parameter interactions would be unlikely to reverse the favorable cost-effectiveness profile we observed.

That said, we recognize the value of such analyses for completeness and have added this point to our limitations section. We note that while our extensive one-way sensitivity analyses support the robustness of our findings, future studies could explore parameter interactions through probabilistic sensitivity analysis to provide additional confirmation of these results.

## Response Letter to Reviewer #2

**Dear Reviewer #2,**

We are deeply grateful for your comprehensive and meticulous review of our manuscript. Your eleven detailed comments have provided invaluable guidance on strengthening our methodological transparency, particularly regarding the specification of clinical trial sources, model structure justification, cost component breakdown, and the generalizability of our findings across China's diverse healthcare settings. We genuinely appreciate the time and expertise you have devoted to improving the rigor and clinical relevance of our work. We have carefully considered each point and made substantial revisions to the manuscript accordingly. Below we provide point-by-point responses to your comments.

## Comment 1

*****Reviewer's Comment:*****

*****The authors should reconsider the change of conclusion for general readership from the viewpoint of this journal.*****

****Response:**** We thank the reviewer for this suggestion. We have revised the conclusion to enhance accessibility for general readership while maintaining scientific rigor. The revised text now emphasizes practical clinical and policy implications in more accessible language, highlighting that PFO closure provides both better health outcomes and lower costs compared to medical therapy alone.

****Revision (Lines 528-535):****

****Original:****

This analysis provides robust evidence that PFO closure represents a dominant strategy for secondary stroke prevention in appropriately selected Chinese patients with cryptogenic stroke. The combination of superior clinical outcomes and cost savings, sustained across comprehensive sensitivity analyses, supports expanded access to PFO closure services within China's healthcare system and should inform clinical practice guidelines and reimbursement decisions.

****Revised:****

In the Chinese healthcare context, percutaneous PFO closure is economically dominant over medical therapy alone for secondary stroke prevention in adults with cryptogenic stroke and confirmed PFO. Patients receiving PFO closure gained 1.41 additional quality-adjusted life years while reducing lifetime costs by $4,045, with 94.2% probability of cost-effectiveness at China's willingness-to-pay threshold. These robust findings support broader implementation of PFO closure programs and provide evidence for clinical practice guidelines and reimbursement policy decisions within national stroke prevention initiatives.

## Comment 2

*****Reviewer's Comment:*****

*****The model uses transition probabilities from "contemporary clinical trials." Could the authors specify exactly which trials (e.g., RESPECT, CLOSE, REDUCE, etc.) were the primary source for the recurrent stroke rates and long-term event probabilities? Furthermore, were these probabilities adjusted for the Chinese patient population, and if so, what was the methodology for that adjustment?*****

****Response:**** We thank the reviewer for this important methodological question.

As detailed in the revised Methods section (Clinical Parameters), transition probabilities for recurrent stroke events were primarily extracted from the RESPECT trial's extended follow-up data [5]. We selected RESPECT over other landmark trials including CLOSE [6] and REDUCE [7] for several reasons: (1) it provides the longest follow-up period (median 5.9 years) among positive trials, yielding more stable long-term event estimates; and (2) the population characteristics (mean age 46 years) closely match our target population. The 46% relative risk reduction observed in our model is consistent with efficacy signals from all three trials, as well as meta-analytic estimates showing 58% reduction in appropriately selected patients [8].

Regarding adjustment for the Chinese population, we employed a multi-faceted approach: (1) stroke severity distribution was directly obtained from Chinese stroke registry data [13]; (2) age-specific background mortality rates were derived from the China Health Statistical Yearbook 2021 [20]; and (3) we conducted sensitivity analyses varying the relative risk reduction from 0.35 to 0.70 (base case 0.53) to account for potential heterogeneity in treatment effects across populations. PFO closure remained cost-effective across this entire range (Table 5).

****Revision (Lines 174-185):****

****Original:****

Transition probabilities for recurrent stroke events were extracted from the RESPECT trial's extended follow-up data [5]. Annual recurrent stroke rates were 0.58 per 100 patient-years for PFO closure and 1.07 per 100 patient-years for medical therapy alone. These annual probabilities were converted to 3-month transition probabilities using the standard formula: P(3-month) = 1 - exp(-rate × 0.25) [17].

Stroke severity distribution was obtained from Chinese stroke registry data, with 53.4% classified as minor stroke (mRS ≤2), 32.4% as moderate stroke (mRS 3-5), and 14.2% resulting in death [13]. Age-specific background mortality rates were derived from the China Health Statistical Yearbook 2021 [18].

****Revised:****

Transition probabilities for recurrent stroke events were extracted from the RESPECT trial's extended follow-up data [5], which served as the primary efficacy source for this analysis. Three landmark randomized controlled trials have demonstrated PFO closure efficacy, including RESPECT [5], CLOSE [6], and REDUCE [7]. We selected RESPECT over other available trials for several reasons. It provides the longest follow-up period (median 5.9 years) among positive trials, yielding more stable long-term event estimates. The population characteristics closely match our target population. Annual recurrent stroke rates were 0.58 per 100 patient-years for PFO closure and 1.07 per 100 patient-years for medical therapy alone, representing a 47% relative risk reduction. These annual probabilities were converted to 3-month transition probabilities using the standard formula: P(3-month) = 1 - exp(-rate × 0.25) [19].

## Comment 3

*****Reviewer's Comment:*****

*****The model includes stable, post-minor recurrent stroke, post-moderate recurrent stroke, and death. Why was a state for post-major/severe recurrent stroke not included? Given that severe stroke can significantly impact costs and QALYs, how does the exclusion of this state potentially bias the cost-effectiveness ratio (ICER)?*****

****Response:**** We thank the reviewer for this important question.

Our "moderate stroke" category (mRS 3-5) encompasses the full spectrum of functional dependence, including what the reviewer refers to as "major/severe stroke" (mRS 4-5 represents severe disability requiring substantial assistance or complete dependence). This three-tier classification (mRS 0-2, mRS 3-5, death) aligns with the modified Rankin Scale's natural division into functional independence, functional dependence, and death, and is consistent with Chinese stroke registry reporting standards [13] and established pharmacoeconomic modeling approaches.

Regarding potential bias, this grouping likely produces conservative estimates of PFO closure benefits. Since PFO closure prevents more strokes overall, and severe strokes (mRS 4-5) incur disproportionately higher costs and lower utilities than mRS 3 strokes, a more granular model separating these categories would likely demonstrate even greater economic benefits for PFO closure. Our sensitivity analyses varied post-stroke utility decrements and long-term care costs across wide ranges (Table 5), effectively capturing the uncertainty associated with stroke severity distribution, and PFO closure remained dominant across all tested scenarios.

We have revised the manuscript to clarify this methodological choice.

****Revision (Lines 120-128):****

****Original:****

The model incorporated four mutually exclusive health states: stable, post-recurrent minor stroke (modified Rankin Scale [mRS] ≤2), post-recurrent moderate stroke (mRS 3-5), and death (Fig 1). The model did not include transitions from post-stroke states back to the stable state.

****Revised:****

The model incorporated four mutually exclusive health states: stable, post-recurrent minor stroke (modified Rankin Scale [mRS] ≤2), post-recurrent moderate stroke (mRS 3-5), and death (Fig 1). **The moderate stroke category encompasses the full spectrum of functional dependence, including severe disability (mRS 4-5), consistent with Chinese stroke registry reporting standards [13]; this grouping likely produces conservative cost-effectiveness estimates, as more granular modeling of severe outcomes would capture their disproportionately higher costs and lower utilities.** The model did not include transitions from post-stroke states back to the stable state.

## Comment 4

*****Reviewer's Comment:*****

*****Please detail the source of the utility values (quality-of-life weights) used for each health state. Were these values derived from Chinese population studies using tools like the EQ-5D, or were they adopted from Western literature? If the latter, how was the transferability to the Chinese context validated?*****

****Response:**** We thank the reviewer for this important question.

As detailed in the revised manuscript (Utility Parameters section), health state utilities were derived from a combination of Chinese and Western sources:

****Post-stroke utilities (Chinese-derived):**** Utility values for post-minor stroke (0.76), post-moderate stroke (0.21), and recurrent stroke events (0.20) were derived from Chinese studies using the EQ-5D instrument with Chinese value sets [25-27]. These values directly reflect health-related quality of life in Chinese stroke populations.

****Stable state utilities (partially Western-derived):**** For stable health states, the baseline utility for medical therapy (0.80) was derived from Chinese stroke survivor data. However, the utility increments following PFO closure (0.84 at 6 months, 0.88 thereafter) were based on Western studies reporting reduced stroke-related anxiety and improved quality of life following successful closure [28].

Regarding transferability validation for the Western-derived utility increments, we employed several approaches:

- We applied the increments as relative changes rather than absolute values, which are generally more transferable across populations
- We conducted extensive sensitivity analyses varying utility parameters across wide ranges (Table 5), with ICERs remaining favorable (-$1,892 to -$3,456 per QALY) when post-stroke utility decrements varied from 0.10 to 0.25
- We explicitly acknowledged this limitation in the manuscript

Importantly, our conclusions are robust to utility assumptions because the economic advantage of PFO closure derives primarily from prevented strokes (and their associated costs) rather than utility improvements. This is evidenced by PFO closure remaining dominant even under conservative utility assumptions.

We have further clarified the utility sources in the revised manuscript.

****Revised (Lines 257-274):****

Health state utilities (numerical values representing health-related quality of life on a scale from 0 for death to 1 for perfect health) were derived from Chinese studies evaluating quality of life in stroke populations (Table 2). Post-stroke utilities were measured using the EQ-5D-3L instrument with Chinese value sets, ensuring cultural and preference consistency with our analysis perspective. Utility values were 0.76 for post-minor stroke, 0.21 for post-moderate stroke, and 0.20 for recurrent stroke events [25-27]. For stable health states, utilities were 0.80 for medical therapy, 0.84 for the initial 6 months following PFO closure, and 0.88 thereafter. These utility increments were based on Western studies reporting reduced stroke-related anxiety and improved quality of life following successful closure [28]. These increments were applied as relative changes to baseline utility, an approach that enhances cross-cultural transferability. We acknowledge the absence of direct utility data from Chinese PFO closure populations. Sensitivity analysis on utility parameters (Table 5) demonstrated that PFO closure remained cost-effective across all tested ranges, with ICERs from -$1,892 to -$3,456 per QALY when post-stroke utility decrement varied from 0.10 to 0.25. This confirms that our conclusions are robust to utility assumptions, as the economic advantage derives primarily from prevented strokes rather than utility improvements.

## Comment 5

*****Reviewer's Comment:*****

*****A 30-year horizon is used. Given that the PFO closure procedure itself is a one-time cost with potentially lifelong benefits, how did the authors ensure that the long-term compliance and sustained efficacy of both PFO closure and medical therapy were accurately modeled over this extended period? Specifically, are the initial relative risk reductions from trials maintained throughout the 30 years, and is this assumption justified?*****

****Response:**** ****Response:****

We thank the reviewer for this important question regarding our long-term modeling assumptions.

****Time horizon justification:**** The 30-year time horizon was selected based on our target population's characteristics. Patients enter the model at mean age 45 years, and a 30-year horizon extends analysis to age 75. According to China national life tables, remaining life expectancy at age 45 is approximately 35 years for the general population. However, patients with prior cryptogenic stroke experience reduced life expectancy due to elevated long-term cardiovascular mortality [17]. We therefore considered 30 years an appropriate proxy for lifetime analysis.

****Sustained efficacy assumptions:****

****PFO closure:**** Sustained efficacy was assumed throughout the model horizon based on the following justifications: (a) PFO closure achieves permanent anatomical correction through device endothelialization, typically complete within 6 months; (b) the RESPECT extended follow-up (median 5.9 years) demonstrated maintained benefit with no evidence of efficacy waning [5]; and (c) the mechanism of benefit, elimination of right-to-left shunt is permanent once successful closure is achieved.

****Medical therapy:**** The base-case analysis conservatively assumed full adherence to antiplatelet/anticoagulant therapy. However, we acknowledge that real-world medication adherence typically declines over time. Our sensitivity analysis varying the relative risk reduction for medical therapy (range: 0.35-0.70) effectively captures potential adherence-related efficacy attenuation.

****Sensitivity analyses addressing long-term uncertainty:**** To validate the robustness of our findings across different time horizons and efficacy assumptions, we conducted comprehensive sensitivity analyses:

- Time horizon sensitivity analysis (10 years to lifetime): PFO closure remained dominant across all horizons tested, with ICERs ranging from -$1,567/QALY at 10 years to -$4,234/QALY at lifetime (Table 5)
- Relative risk reduction sensitivity analysis (0.35-0.70): PFO closure remained cost-effective across all tested values, with ICERs ranging from dominant to -$892/QALY

These analyses confirm that our conclusions are robust to uncertainty in long-term efficacy assumptions.

We have added a new subsection "Long-term Efficacy Assumptions" in the Methods section to clarify these modeling assumptions .

****Add the following text (Lines 244-255):****

****Long-term Efficacy Assumptions****

Long-term efficacy assumptions were specified as follows. For PFO closure, sustained efficacy was assumed throughout the model horizon based on permanent anatomical correction achieved by the procedure; device endothelialization is typically complete within 6 months, and the RESPECT extended follow-up demonstrated maintained benefit with no evidence of efficacy waning [5]. For medical therapy, the base-case analysis conservatively assumed full adherence to antiplatelet/anticoagulant therapy. However, real-world medication adherence typically declines over time; sensitivity analysis varying the relative risk reduction for medical therapy (range: 0.35-0.70) was conducted to account for potential adherence-related efficacy attenuation, and PFO closure remained cost-effective across all tested values.

## Comment 6

*****Reviewer's Comment:*****

*****Since the analysis is from the Chinese healthcare payer perspective, what specific costs were included (e.g., procedure costs, hospitalization, initial and long-term medication, device/implant cost, stroke rehabilitation, long-term care)? Were indirect costs (like lost productivity), which can be substantial, excluded, and if so, how might their inclusion affect the dominance finding?*****

****Response:****

We thank the reviewer for this important question regarding cost components.

From the Chinese healthcare payer perspective, the analysis included comprehensive direct medical costs as detailed in Table 1 and the Methods section. PFO closure costs comprised device expenses ($4,938 for Amplatzer PFO Occluder), procedural costs ($517), and associated hospitalization costs ($1,180), totaling $6,635 per procedure. Acute stroke management costs were $1,901 for minor stroke (mRS ≤2) and $2,513 for moderate stroke (mRS 3-5). Long-term post-stroke care costs, encompassing rehabilitation services, outpatient follow-up, and ongoing medical management, were modeled as quarterly costs of $338 for post-minor stroke care and $514 for post-moderate stroke care. Antithrombotic medication costs included aspirin ($0.80/month), clopidogrel ($8.50/month), and warfarin ($2.30/month). Additionally, psychological comorbidity-related medical costs were included as specified in Table 1.

Consistent with the healthcare payer perspective, indirect costs including productivity losses, informal caregiving, and transportation were excluded from the base-case analysis. However, we believe this exclusion makes our findings conservative rather than overstating the economic advantage of PFO closure. Cryptogenic stroke predominantly affects working-age adults (mean age 45 years in our model), for whom productivity losses from recurrent stroke would be substantial. Since PFO closure prevents more strokes than medical therapy alone (0.15 fewer strokes per patient over 30 years), including indirect costs would increase the cost savings associated with PFO closure, thereby strengthening the dominance finding rather than reversing it. This interpretation is supported by our sensitivity analyses, which demonstrated that PFO closure remained economically dominant even when direct costs were varied substantially across all parameters.

We have added clarifying text to the Methods section explicitly stating the exclusion of indirect costs and its implications . We have also acknowledged this limitation in the Discussion section and identified societal perspective analysis as a future research priority.

****Add the following text (Lines 238-243):****

Consistent with the healthcare payer perspective, indirect costs including productivity losses and informal caregiving were excluded from the analysis. This exclusion likely makes our findings conservative, as cryptogenic stroke predominantly affects working-age adults for whom productivity losses from recurrent stroke would be substantial; including such costs would strengthen rather than diminish the economic advantage of PFO closure.

## Comment 7

*****Reviewer's Comment:*****

*****What is the breakdown of the initial PFO closure cost? Specifically, what cost was assumed for the PFO closure device itself, and does this cost reflect actual procurement prices negotiated by Chinese hospitals or a general market price?*****

****Response:****

We thank the reviewer for this question regarding the PFO closure cost structure.

The breakdown of initial PFO closure costs is presented in Table 1 and comprises three components: device cost ($4,938, range $3,703-6,173), procedure cost ($517, range $388-646), and hospitalization and other costs ($1,180, range $885-1,475), totaling $6,635 per procedure (range $4,976-8,294). The device cost specifically reflects the Amplatzer PFO Occluder, which is the most commonly used closure device in China and was employed in the pivotal RESPECT trial that informed our efficacy parameters.

These cost estimates reflect actual procurement prices negotiated by Chinese hospitals rather than general market or list prices. We conducted cost surveys across multiple Chinese tertiary hospitals with established PFO closure programs to ensure representativeness. The device cost of $4,938 represents the median negotiated procurement price reported by surveyed institutions, with the range ($3,703-6,173) capturing inter-hospital variation in negotiated prices across different provinces and hospital tiers. This approach was chosen because device prices in China vary substantially based on provincial tendering processes, hospital purchasing volume, and negotiation with distributors.

To address uncertainty in device pricing, we conducted sensitivity analyses varying total PFO closure procedure costs. Sensitivity analysis increasing procedure costs by 20% confirmed that PFO closure remained dominant, demonstrating that our conclusions are robust to potential variations in device procurement prices across different Chinese healthcare settings.

We have added clarifying text to the Methods section explicitly stating that prices reflect actual negotiated procurement prices.

****Current text:****

All costs were expressed in 2021 US dollars from a Chinese healthcare payer perspective (Table 1). PFO closure costs included device expenses ($4,938 for Amplatzer PFO Occluder), procedural costs ($517), and associated hospitalization costs ($1,180), totaling $6,635 per procedure. These estimates were obtained from cost surveys across multiple Chinese tertiary hospitals to ensure representativeness.

****Revised text (Lines 200-207):****

All costs were expressed in 2021 US dollars from a Chinese healthcare payer perspective (Table 1). PFO closure costs included device expenses ($4,938 for Amplatzer PFO Occluder), procedural costs ($517), and associated hospitalization costs ($1,180), totaling $6,635 per procedure. These estimates were obtained from cost surveys across multiple Chinese tertiary hospitals to ensure representativeness. The device cost reflects actual negotiated procurement prices rather than list prices, with the range ($3,703-6,173) capturing inter-hospital variation across different provinces and hospital tiers.

## Comment 8

*****Reviewer's Comment :*****

*****The results state that psychological comorbidity costs exerted a great influence on the results. Could the authors elaborate on the nature and magnitude of these costs? How were they measured, and why do they have such a disproportionate impact on the model's output?*****

****Response to Reviewer****

We thank the reviewer for this insightful question regarding psychological comorbidity costs.

****Nature and Magnitude of Costs:****

Psychological comorbidity costs in our model encompass healthcare expenditures associated with depression, anxiety, and psychological distress following cryptogenic stroke. As shown in Table 1, we estimated quarterly costs of $125 (range $94-156) for depression/anxiety and $89 (range $67-111) for high psychological distress states. These costs include outpatient psychiatric consultations, psychotropic medications (antidepressants, anxiolytics), and psychological counseling services.

****Measurement:****

The estimates were derived from expert clinical opinion based on typical treatment patterns for post-stroke psychological complications in Chinese healthcare settings, as published Chinese-specific data on these costs were limited.

****Why These Costs Have Disproportionate Impact:****

The substantial influence of psychological comorbidity costs on model outputs stems from three factors:

****High prevalence:**** Post-stroke depression affects 30-50% of stroke survivors, and anxiety disorders occur in approximately 20-25% of patients. Our model incorporated these evidence-based prevalence rates, meaning a substantial proportion of the cohort incurs these costs.

****Cumulative effect over time:**** Unlike one-time procedural costs, psychological comorbidity costs are recurring quarterly expenses that accumulate over the lifetime horizon. Over a 40-year time horizon, quarterly costs of $125 translate to $500 annually, potentially exceeding $20,000 over the model horizon for affected patients.

****Differential impact between arms:**** Patients experiencing recurrent stroke have higher rates of psychological comorbidity. Since PFO closure reduces recurrent stroke risk, it consequently reduces the proportion of patients incurring these ongoing psychological costs, creating a sustained cost advantage for the PFO closure arm that amplifies over time.

Importantly, our sensitivity analyses confirmed that even when psychological comorbidity costs were varied across their full ranges or set to zero, PFO closure remained cost-effective, indicating that our conclusions are not dependent on their precise values.

We have added clarifying text to the Methods section to elaborate on these costs.

****Add the following text (Lines 225-237):****

Psychological comorbidity costs were estimated based on expert clinical opinion, as published Chinese-specific data were limited (Table 1). Quarterly costs of $125 (range $94-156) for depression/anxiety and $89 (range $67-111) for high psychological distress include outpatient psychiatric consultations, psychotropic medications, and psychological counseling services. These costs exert substantial influence on model outputs due to three factors: high prevalence of post-stroke psychological complications (depression affects 30-50% of stroke survivors), cumulative effect of recurring quarterly costs over the lifetime horizon, and differential impact between treatment arms. Since PFO closure reduces recurrent stroke risk, it consequently reduces the proportion of patients incurring these ongoing costs, creating a sustained cost advantage that amplifies over time. Sensitivity analyses confirmed that PFO closure remained cost-effective even when psychological costs were varied across their full ranges.

## Comment 9

*****Reviewer's Comment :*****

*****The PSA showed a 94.2% probability of cost-effectiveness. While this is high, could the authors provide the corresponding Cost-Effectiveness Acceptability Curve (CEAC)? This visual representation would be very helpful to see the probability of cost-effectiveness across a range of willingness-to-pay thresholds, not just the single stated threshold.*****

****Response to Reviewer****

We thank the reviewer for this suggestion. We would like to clarify that the Cost-Effectiveness Acceptability Curve (CEAC) was included in our original submission as **Figure 3**. We apologize if this was not sufficiently highlighted in the text.

To address the reviewer's concern and provide a more comprehensive interpretation, we have expanded the description of the CEAC in the Results section. The CEAC demonstrates that PFO closure has a high probability of being cost-effective across a wide range of WTP thresholds:

- At a WTP threshold of $0/QALY, PFO closure has an 87.3% probability of being dominant (i.e., cost-saving and more effective), indicating that in the majority of simulations, PFO closure both reduces costs and improves health outcomes.
- At 1.5× GDP per capita ($18,827/QALY), the probability is 91.8%.
- At the Chinese threshold of 3× GDP per capita ($37,654/QALY), the probability reaches 94.2%.
- The curve plateaus at approximately 95% at higher WTP thresholds.

These results confirm that our findings are robust across different WTP thresholds and support the conclusion that PFO closure represents excellent value for money in the Chinese healthcare context.

The Results section has been revised accordingly (Probabilistic Sensitivity Analysis section).

****Current text:****

Monte Carlo simulation with 10,000 iterations confirmed the robustness of base-case findings. The cost-effectiveness acceptability curve demonstrated that PFO closure had a 94.2% probability of being cost-effective at China's willingness-to-pay threshold of $37,654 per QALY. At lower thresholds of $18,827 per QALY, representing 1.5 times per capita GDP, the probability remained high at 91.8%.

****Revised text (Lines 379-388):****

Monte Carlo simulation with 10,000 iterations confirmed the robustness of base-case findings. The cost-effectiveness acceptability curve (Figure 3) illustrates the probability of PFO closure being cost-effective across a range of WTP thresholds. At a WTP of $0/QALY, PFO closure demonstrated an 87.3% probability of being dominant (both cost-saving and more effective), reflecting its strong economic profile even without considering willingness-to-pay for health gains. The probability of cost-effectiveness increased to 91.8% at 1.5× GDP per capita ($18,827/QALY) and reached 94.2% at China's threshold of 3× GDP per capita ($37,654/QALY). The curve plateaus at approximately 95% at higher thresholds, indicating robust cost-effectiveness across different decision-making contexts.

## Comment 10

*****Reviewer's Comment:*****

*****How do the authors expect these results to generalize across different tiers of hospitals or regions within China (e.g., tertiary hospitals in major cities vs. county-level hospitals), where costs, expertise, and patient profiles may vary significantly?*****

****Response to Reviewer****

We thank the reviewer for raising this important question regarding the generalizability of our findings across China's diverse healthcare landscape.

****Expanded sensitivity analysis:****

To address concerns about cost variability across different hospital tiers and regions, we have expanded our sensitivity analysis to include ****±75% variation in PFO closure procedure costs**** (range: $1,659 to $11,611, compared to the base case of $6,635). This wide range encompasses the potential cost differences between:

- Lower-cost settings (e.g., high-volume tertiary centers with economies of scale)
- Higher-cost settings (e.g., centers with lower procedural volumes, premium device pricing, or additional diagnostic requirements)

****Key findings supporting generalizability:****

As shown in the updated Table 5, PFO closure remained ****dominant (cost-saving and more effective)**** across the entire ±75% cost range, with ICERs ranging from -$5,038 to -$698 per QALY. This demonstrates that:

1.Even when PFO closure costs increase by 75% to $11,611 (simulating higher costs at less experienced centers or regions with elevated pricing), the intervention remains cost-saving (ICER: -$698/QALY).

2.At lower costs ($1,659, representing high-volume centers or future price reductions), the economic advantage becomes even more pronounced (ICER: -$5,038/QALY).

3.No threshold was identified at which PFO closure would become cost-ineffective, indicating robust economic conclusions across all plausible cost scenarios.

****Additional considerations:****

We acknowledge that our base-case analysis primarily reflects tertiary hospital settings where PFO closure is currently performed. Important considerations for generalizability include:

- ****Expertise and outcomes:**** Procedural success rates and complication rates may vary with operator experience. However, our sensitivity analysis on procedure-related mortality (0.0%-0.5%) showed robust cost-effectiveness across this range.
- ****Patient selection:**** Appropriate patient selection requires specialized diagnostic capabilities (TEE, bubble study) that may be limited in lower-tier hospitals.
- ****Referral pathways:**** In practice, patients from county-level hospitals would likely be referred to tertiary centers for PFO closure, with the procedure costs remaining within the tested range.

****Current text:****

From a policy perspective, these findings support strategic expansion of PFO closure capabilities within China's healthcare infrastructure. The robustness of economic benefits across ±30% regional cost variations suggests broad applicability across diverse provincial reimbursement environments. Integration of structured PFO evaluation and referral pathways could enhance both clinical outcomes and allocative efficiency within national stroke prevention initiatives.

****Revised text (Lines 479-491):****

From a policy perspective, these findings support strategic expansion of PFO closure capabilities within China's healthcare infrastructure. The robustness of economic benefits across ±75% variation in procedural costs suggests broad applicability across different hospital tiers and regional reimbursement environments. Notably, PFO closure remained cost-saving even when procedural costs were increased by 75% to $11,611, indicating that the economic advantage would persist across tertiary hospitals in major cities as well as settings with higher operational costs or lower procedural volumes. While PFO closure is currently concentrated in tertiary hospitals, the establishment of structured referral pathways from lower-tier hospitals could extend these economic benefits to patients across all regions. Integration of structured PFO evaluation and referral pathways could enhance both clinical outcomes and allocative efficiency within national stroke prevention initiatives.

## Comment 11

*****Reviewer's Comment :*****

*****I will recommend authors to use ([https://doi.org/10.3390/scipharm93010006](https://doi.org/10.3390/scipharm93010006) and [https://doi.org/10.1093/narmme/ugae005](https://doi.org/10.1093/narmme/ugae005) and [https://doi.org/10.1002/ange.202215245](https://doi.org/10.1002/ange.202215245)) in addition to their references.*****

****Response to Reviewer****

We sincerely thank the reviewer for taking the time to suggest additional references and for the effort in identifying literature that might enhance our manuscript. Following the Academic Editor's guidance to "carefully evaluate any literature suggested for citation" and to "only include references that are scientifically relevant," we have thoroughly and respectfully reviewed all three recommended publications:

1.Thumpati P, et al. (2025) "Targeting c-MYC G-Quadruplexes for Cancer Treatment with Small Molecules." Scientia Pharmaceutica. DOI: 10.3390/scipharm93010006

2.Chaudhuri R, et al. (2024) "Combating multidrug-resistance in S. pneumoniae: a G-quadruplex binding inhibitor of efflux pump and its bio-orthogonal assembly." NAR Molecular Medicine. DOI: 10.1093/narmme/ugae005

3.Chaudhuri R, et al. (2022) "Expanding the Toolbox of Target Directed Bio-Orthogonal Synthesis: In Situ Direct Macrocyclization by DNA Templates." Angewandte Chemie. DOI: 10.1002/ange.202215245

We recognize that these are valuable contributions to the fields of G-quadruplex DNA structures, cancer therapeutics, antimicrobial resistance, and bio-orthogonal chemistry. However, after careful consideration, we found it challenging to identify a direct connection between these research areas and our health economic evaluation of PFO closure for cryptogenic stroke prevention. As our study primarily addresses cardiovascular intervention outcomes, Markov modeling, and cost-effectiveness methodology, we were unable to establish a clear scientific linkage that would justify their inclusion.

We have made every effort to ensure that our reference list comprehensively covers the relevant literature in health technology assessment, PFO closure clinical evidence, stroke epidemiology, and pharmacoeconomic methodology. We hope the reviewer understands our consideration, and we would be most grateful if the reviewer could kindly point out any specific aspects of our manuscript where these references might be applicable, or alternatively suggest other references within the cardiovascular or health economics domains that could further strengthen our work.

We greatly appreciate the reviewer's continued guidance and support in improving our manuscript.

.
