# Supplementary material for: Cost-effectiveness of percutaneous patent foramen ovale closure versus medical therapy for cryptogenic stroke prevention: A Chinese healthcare perspective
Source: PLoS One. 2026 Mar 17;21(3):e0345015. doi: 10.1371/journal.pone.0345015 (PMC12994829; doi:10.1371/journal.pone.0345015)
Supplement: Attachment — Submitted filename: Response to Editor.docx [file pone.0345015.s009.docx]

**Response to Academic Editor and Reviewer**

**Manuscript ID:** PONE-D-25-45858R1

**Title:** Cost-Effectiveness of Percutaneous Patent Foramen Ovale Closure versus Medical Therapy for Cryptogenic Stroke Prevention: A Chinese Healthcare Perspective

**Journal:** PLOS ONE

**Date:** February 2026

Dear Dr. Jeerath Phannajit,

Thank you for the constructive review of our manuscript. We also appreciate Reviewer #1’s positive assessment and recommendation for publication. We have carefully addressed each of the six points you raised regarding data consistency. Below we provide point-by-point responses to both the Academic Editor and Reviewer #1, along with a summary of all changes made.

**Comment 1:**

**Editor’s comment:** *“Please reconcile the base-case discounting assumption because the manuscript Methods (‘Model structure / time horizon and discounting’) states that costs and outcomes were discounted at 5% annually (line 159), but the one-way sensitivity analysis table uses 3% as the base-case discount rate (Table 5).”*

**Response:** We thank the editor for identifying this typographical error. The base-case discount rate used in our model was indeed 5% annually, consistent with the China Guidelines for Pharmacoeconomic Evaluations [18]. The “3%” that appeared in Table 5 was a transcription error introduced during a previous revision. We have now corrected Table 5 to show the base-case discount rate as 5%, with a sensitivity analysis range of 3%–7%. The range of 3%–7% was selected to encompass the discount rates commonly recommended by international pharmacoeconomic guidelines (e.g., 3% as widely adopted in global cost-effectiveness analyses and 5% by the China Guidelines), which is also consistent with the tornado diagram (Figure 2, which labels the parameter as “Discount rate (3%–7%)”). Importantly, all model results were generated using the correct 5% discount rate and remain unchanged.

**Changes made:** Table 5, “Discount rate” row: Base Case corrected from “3%” to “5%”; Range corrected from “0%–6%” to “3%–7%”.

**Comment 2:**

**Editor’s comment:** *“Please recheck the price year because the Methods (‘Cost Parameters’) states all costs were expressed in 2021 USD (line 195), while the base-case results table 3 is labeled as USD (2023).”*

**Response:** We apologize for this labeling error. All costs in our analysis were expressed in 2021 USD, as stated in the Methods section (line 195) and the Table 1 footnote (“All costs expressed in 2021 USD from Chinese healthcare payer perspective”). No Consumer Price Index adjustment was performed. The “2023” label in the Table 3 column header was a typographical error. We have corrected it to “USD, 2021.” The underlying cost data and model results are unchanged.

**Changes made:** Table 3, column header: “Total Costs (USD, 2023)” corrected to “Total Costs (USD, 2021)”.

**Comment 3:**

**Editor’s comment:** *“Please resolve the mismatch where acute stroke management costs in the Methods (page 20, line 213) and Table 1 are around $1,901 (minor) and $2,513 (moderate), but Table 5 lists ‘Stroke treatment cost (acute)’ as $28,500 (range $20,000–$40,000).”*

**Response:** We sincerely thank the editor for catching this significant discrepancy. The $28,500 value in Table 5 was erroneous and did not reflect the actual model inputs. The correct acute stroke management costs used in our model were $1,901 for minor stroke (mRS 0–2) and $2,513 for moderate-to-severe stroke (mRS 3–5), exactly as reported in the Methods section and Table 1.

We have corrected Table 5 by splitting this into two separate severity-specific parameters, with base-case values taken directly from Table 1 and sensitivity ranges set at ±20% of base case:

• Acute stroke management cost, minor (mRS 0–2): Base case $1,901, Range $1,521–$2,281, ICER range −2,745 to −2,990

• Acute stroke management cost, moderate-to-severe (mRS 3–5): Base case $2,513, Range $2,010–$3,016, ICER range −2,760 to −2,976

Similarly, the “Long-term stroke care cost” row ($12,000) did not correspond to the quarterly values reported in Table 1 ($338 for post-minor stroke and $514 for post-moderate-to-severe stroke). We have corrected this by splitting it into two separate severity-specific parameters, with base-case values from Table 1 and sensitivity ranges set at ±25% to reflect greater uncertainty in long-term care estimates:

• Ongoing post-stroke care cost, minor (quarterly): Base case $338, Range $254–$422, ICER range −2,275 to −3,462

• Ongoing post-stroke care cost, moderate-to-severe (quarterly): Base case $514, Range $386–$643, ICER range −2,214 to −3,523

The ICER ranges for these parameters remain consistent with PFO closure maintaining cost-effectiveness across all tested values.

**Changes made:** Table 5: (1) “Stroke treatment cost (acute)” row replaced with two rows: “Acute stroke management cost, minor (mRS 0–2)” (base case $1,901; range $1,521–$2,281; ICER range −2,745 to −2,990) and “Acute stroke management cost, moderate-to-severe (mRS 3–5)” (base case $2,513; range $2,010–$3,016; ICER range −2,760 to −2,976). (2) “Long-term stroke care cost” row replaced with two rows: “Ongoing post-stroke care cost, minor (quarterly)” (base case $338; range $254–$422; ICER range −2,275 to −3,462) and “Ongoing post-stroke care cost, moderate-to-severe (quarterly)” (base case $514; range $386–$643; ICER range −2,214 to −3,523).

**Comment 4:**

**Editor’s comment:** *“Please make sure aspirin dose consistent because Table 1 lists aspirin 75 mg daily, but the Methods text lists aspirin 100 mg daily.”*

**Response:** Thank you for noting this inconsistency. The correct aspirin dose used in our analysis is 100 mg daily, which reflects the standard secondary stroke prevention dose recommended in Chinese clinical practice guidelines. The “75 mg” in Table 1 was a typographical error. We have corrected Table 1 to read “Aspirin 100mg daily.” The unit cost ($0.80/month) corresponds to the 100 mg daily formulation and remains unchanged.

**Changes made:** Table 1: “Aspirin 75mg daily” corrected to “Aspirin 100mg daily.”

**Comment 5:**

**Editor’s comment:** *“Please consider use term moderate-to-severe stroke instead of moderate stroke consistently across the manuscript and tables, because the text explicitly states the category includes severe disability (mRS 4–5).”*

**Response:** We agree with the editor’s suggestion. Since our “moderate stroke” category encompasses mRS 3–5, which includes severe disability states (mRS 4–5), the term “moderate-to-severe stroke” is more clinically accurate. We have systematically replaced “moderate stroke” with “moderate-to-severe stroke” throughout the manuscript.

**Changes made:**

• Manuscript text: all instances updated.

• Table 1: “Moderate stroke (mRS 3-5)” → “Moderate-to-severe stroke (mRS 3-5)”; “Post-moderate stroke care” → “Post-moderate-to-severe stroke care.”

• Table 2: “Post-moderate stroke (mRS 3-5)” → “Post-moderate-to-severe stroke (mRS 3-5).”

• Table 4: “Moderate strokes (mRS 3-5)” → “Moderate-to-severe strokes (mRS 3-5)”; footnote updated accordingly.

• Figure 1: “Post-recurrent moderate stroke” → “Post-recurrent moderate-to-severe stroke.”

**Comment 6**

**Editor’s comment:** *“Results table should align with stated cost year and discounting assumptions: After harmonizing the price year and discount rate, please re-generate Table 3 so its totals, incremental values, and any dominance/ICER statements are consistent with the corrected assumptions.”*

**Response:** We confirm that our economic model was consistently run using a 5% annual discount rate and 2021 USD throughout the entire analysis. The discrepancies identified by the editor (3% discount rate in Table 5 and “USD 2023” in Table 3) were labeling and transcription errors only; they did not affect the actual model calculations or outputs.

To verify this, we performed cross-validation against two independent figures:

• Figure 2 (Tornado Diagram): The base-case Net Monetary Benefit is annotated as $57,137. This can be independently calculated from Table 3 values: NMB = ΔE × WTP − ΔC = 1.41 × $37,654 − (−$4,045) = $53,092 + $4,045 = $57,137. （√）

• Figure 4 (PSA Scatter Plot): The Mean ICER is annotated as −$2,868/QALY, which equals ΔC/ΔE = −$4,045/1.41 = −$2,868. （√）

Both figures were generated directly from the model and confirm that Table 3 values are correct and internally consistent. Therefore, Table 3 does not require recalculation. We have corrected the column header to accurately reflect the price year (USD, 2021).

**Changes made:** Table 3 column header corrected (as detailed in Comment 2). Table 5 discount rate corrected (as detailed in Comment 1). Table 5 cost parameters corrected and split into severity-specific rows (as detailed in Comment 3). Cross-validation with Figures 2 and 4 confirmed all base-case numerical results in Table 3 are internally consistent and unaffected by these corrections.

**Response to Reviewer #1**

We sincerely thank Reviewer #1 for the positive and encouraging evaluation of our manuscript. We are grateful for the recommendation for publication and the time dedicated to reviewing our work. As noted by the reviewer, all comments from the previous round of review have been addressed. In the current revision, we have focused on resolving the data consistency issues identified by the Academic Editor, as detailed above.

**Summary of All Changes**

| **Issue** | **Location** | **Change** |
| --- | --- | --- |
| Discount rate | Table 5 | Base case: 3% → 5%; Range: 0%–6% → 3%–7% |
| Price year | Table 3 | Column header: USD, 2023 → USD, 2021 |
| Acute stroke cost (minor) | Table 5 | New row: base case $1,901; range $1,521–$2,281 |
| Acute stroke cost (mod-sev) | Table 5 | New row: base case $2,513; range $2,010–$3,016 |
| Post-stroke care (minor) | Table 5 | New row: base case $338; range $254–$422 |
| Post-stroke care (mod-sev) | Table 5 | New row: base case $514; range $386–$643 |
| Aspirin dose | Table 1 | 75mg → 100mg daily |
| Terminology | Throughout | “moderate stroke” → “moderate-to-severe stroke” |
| Figure 1 | State label | Updated to “moderate-to-severe stroke” |

We believe these revisions address all concerns raised by the editor and reviewer. We are grateful for the opportunity to improve the consistency of our manuscript. Please do not hesitate to contact us should any further clarification be needed.
